# Addendum—Herrick on Cholera

**Published:** 1849-07

**Authors:** William B. Herrick

**Affiliations:** Professor of Anatomy in Rush Medical College, Chicago


### CHOLERA,

### *Its Cause, Pathology and Treatment.*

By William B. Herrick, M. D., Professor of Anatomy in Rush Medical College, Chicago.

In making some remarks upon Cholera in the January No. of this Journal, it was stated in substance, as our opinion, that the electrical changes constantly taking place on the earth's surface, in the atmosphere and in our own and the bodies of other animals, must have an influence either directly or indirectly in the modification and production of this and other similar epidemics.

At that time and during the previous fall and winter, my young friend Dr. J. H. Bird and myself had been collecting facts with the view of determining to what extent, the substance produced during the passage of electrical currents through the atmosphere, called by the discoverer *ozone*, might act as a deleterious agent in causing disease.

Our attention was first directed to this subject by reading an extract from a German Periodical, in the Amer. Jour. Med. Sciences for July 1848, in which it is stated in substance that experiments made some years since by Prof. Schonbein, tended to show that where ordinary electricity passes from pointed bodies, through the atmosphere, this substance is produced.

The experiments made by Schonbein, soon after the discovery of *ozone*, were verified by numerous other chemists, in order to determine its true nature and chemical relations. A good description of the different circumstances under which *ozone* is produced naturally, and of the various methods for obtaining it artificially, may be found in an extract from a letter to M. Dumas from M. d'Marignac, published in the Chemical Gazette for April 1845.

Most chemists describe *ozone* as being constituted of three parts of oxygen to one of Hydrogen, making a tritoxide of Hydrogen. All agree that it is one of the most powerful oxidizing agents known. By virtue of this property, it decomposes most compounds constituted in whole or in part of substances having an affinity for oxygen, such as sulphureted and carbureted Hydrogen, Iodide of Potassium, &c., converts protoxides, into peroxides and gives up to carbon and hydrogen its oxygen to form carbonic acid and water. The best test for *ozone* is iodide of potassium dissolved in a solution of starch; the *ozone*, acting as a decomposing agent upon the iodide of potassium, liberates iodine.—Uncombined iodine, having a strong affinity for starch, combines with it, to form the well known compound, iodide of starch, the beautiful blue color of which forms its principal characteristic. In view of these facts, it is easily understood, why it is, that strips of paper moistened with the above named solution and exposed to an atmosphere containing *ozone*, assume a blue tint, more intense, and acquired more rapidly in proportion to the amount of *ozone* present.

The smallest possible quantity of *ozone*, it is said, can be detected by this test; in using it however, care should be taken, not to expose the strips of paper in situations where gases, such as sulphureted and carbureted hydrogen, may be suspected to be present, as these, as well as most other highly combustible substances, decompose *ozone*.

*Ozone*, acting as it does, as an intense oxidizing agent, is known to have a powerful influence both upon organic and inorganic substances, in causing chemical changes. We might be led to infer, therefore, even in the absence of facts to support the conclusion, that animals living in and breathing an atmosphere containing it, would be liable to become in some way affected by its influence. All the facts, and the results of a few experiments, fully justify this conclusion.

Small animals confined in an atmosphere impregnated with *ozone*, die in from five to ten minutes, and two or three inhalations of *ozonized* air by individuals engaged in experiments, have uniformly produced irritation and, in some instances, a degree of inflammation of the mucous membrane lining the air passages, similar to that caused by breathing chlorine or bromine; hence the conclusion arrived at by Schonbein that *ozone* might be the principal, if not, the only cause of epidemic influenza.

These conclusions of Schonbein are well sustained both by arguments and facts drawn from various sources. In the Amer. Jour. of Med. Science for July 1848 may be found the translation from Schonbein's article, from which we take the following brief extract:

"Now if it were shown, that at certain periods, characterized by a general prevalence of catarrhal affections, large quantities of chlorine or bromine were present in the atmosphere, no one would hesitate to ascribe the cause of these diseases to the above substances. But it is an established fact, that by the inhalation of proportionally small quantities of ozone, physiological effects are produced similar to those which are occasioned by the inhalation of air charged with chlorine or bromine. This led Schonbein many years ago to conjecture that many catarrhal affections might be owing to the presence of ozone in the atmosphere. In the course of last winter, several catarrhal epidemics occured in Bâsle, so that very few persons escaped; and Schonbein and many physicians of that town, instituted a series of daily observations, with the view of ascertaining how far the rapidity and intensity of the blue coloration of the iodide paste were connected with the prevalence and intensity of the catarrhal symptoms: the results were conclusive as to the simultaneity of the maximum of the coloration with the extremest intensity of the epidemic."

Dr. Spengler states "that in the village of Roggendorf, in Mecklenburgh, towards the close of 1846, slight catarrhal affections became prevalent,—that but slight trace of ozone was then to be detected in the air. With the opening of the following year, however, these catarrhal affections assumed the severest forms of tracheal and bronchial disease, hooping-cough became common, both among children and adults; then reagents detected a great increase of ozone in the atmosphere, and, at the same time, influenza spread over the district. On the 9th January, the *ozonometer* showed a still further increase in the proportion of ozone present in the air. On the same day two persons died of influenza, and gradually the influenza spread more extensively, until, on the 21st, scarcely an individual had escaped. Thus there seemed a decided connection between the presence of ozone in the air and the spread of the epidemic."

Dr. Day, in a scientific and well written work on the "Diseases of advanced life" recently published, gives a brief history of the epidemic influenza, which prevailed in England in 1847, and in his remarks upon the causes, uses the following language:

"A theory has been recently propounded by an eminent German chemist (Schonbein, the discoverer of gun-cotton) that epidemic influenza is dependent on the presence of an imponderable agent termed *ozone*, in the atmosphere. This ozone is apparently a result of atmospheric electricity. It may be prepared in the laboratory of the chemist, and from what we know of its properties there are *à priori* reasons for suspecting that it would, if existing in the air we breathe, give rise to great irritation of the respiratory organs. Several physicians have actually found that an excess of this material was present in the atmosphere during the late epidemic."

Numerous other facts and arguments might be urged in support of Schonbein's theory, but want of time and space compels us to close our remarks upon this point, with the expression of the belief on our part, that subsequent observations and experiments will ultimately sustain us in what we now assume to be a fact, as well substantiated as any other in medicine of a similar character, that *ozone is the cause of epidemic influenza.*

Having arrived at this important conclusion, we are naturally led to inquire, in the next place, if other and similar diseases may not be dependent upon the same cause? The history of most epidemics shows, that in many respects, they are similar, appearing and disappearing as most of them do, without any assignable cause: severe and fatal in their effects at the onset; but more mild, and comparatively harmless, as the time approaches for their disappearance.

Asiatic Cholera is a disease, the history of which is so nearly allied to that of epidemic influenza, that a description of either, needs but a change of the name and we have an equally correct account of the other, as will be seen by taking the following brief extracts from Dr. Hancock's book on Cholera, published in London in 1832, and comparing them, with others placed opposite, selected from Dr. Day on Epidemic Influenza, contained in his new work on the "Diseases of advanced life."

*Dr. Hancock on Cholera.*

"The cholera traversed the great part of Europe last year (1831.) In Warsaw it appeared in April, Dantzic and Riga, it visited in May; Archangel and Petersburgh, in June; Pesth and Bucharest in July; it reached Berlin in August; Vienna in September; Hamburgh in October; and the eastern shores of England at the close of the year."

*Dr. Day on Epidemic Influenza.*

"The great epidemics (Influenzas) generally travel from Russia, over Germany Denmark, Sweden, England France, Italy, Spain, in from three to six months; and then reach America."

*Those attacked with Cholera* "were the poor, ill fed and wretched; and it broke out in the low, confined and filthy places in which the population was most dense."*

"Influenza appears to be generated in ill organized camps and in crowded, ill cleansed cities, and to be the most fatal among people who have for some time been depressed and ill fed."

"It was severe and generally fatal at the beginning, and mild and manageable at the decline, in every place. Its duration as an epidemic seldom exceeded two months."

"It attained the greatest intensity in the second week of its course; raged with nearly equal violence the third week; declined in the fourth, and then partly disappeared."

The analogy between the two Epidemics under consideration, is shown to be most remarkable, by the following quotations from different authors.

Dr. Hancock states, that at the close of the year 1832, it had appeared on the Eastern coast of England, after having passed over the Northern and Eastern part of Europe. The great part of the West and South of Europe had not as yet been visited by the Epidemic. "But" says he "these parts though exempt from Cholera have not been free from other maladies."

"In France, we are told, that the pulmonary Catarrh or *Grippe*, has been raging to a considerable extent, and has even reached Italy, creating much alarm at Rome. Diseases in truth seem to have experienced remarkable interchanges of climate and season. While Europe has had Cholera as it appeared in India, some parts of India, as Culcutta, have been affected with a disease, which they call a new Epidemic, like the Influenza or *Grippe*, lately prevalent in France. Cholera, a disease of Autumn and heat, has shown itself in the cold of Winter."

Dr. J Spencer, one of the most correct observers, and talented physicians of our own country, wrote an essay on Cholera, which was published during its prevalence here in 1833, from which we take the following extract expressive of his belief, even at that time, in the origin of both diseases from the same cause. "Influenza has generally preceded the invasion of the present epidemic, and presents an interesting association with the history of this malady. The epidemic predisposition in the influenza, appears to be thrown upon the mucous membrane of the lungs, which entirely resembles in its organization the structure of the intestinal membrane."

A comparison of the history of both epidemics shows that influenza has always proceeded, frequently accompanied, and generally followed, the appearance of cholera.

Meteorological facts and statistics show that the state of the atmosphere, as to its electrical condition, temperature, humidity, movements, &c., is similar, previous to the appearance and during the prevalence of Cholera, to that which precedes and accompanies influenza. Influenza, like cholera, finds by far the greater number of its victims in the narrow, badly ventilated streets of large cities, among the half-clothed, starving victims of poverty. During the prevalence of both cholera and influenza, all classes suffer more or less; some to a greater, others to a less extent. Both diseases attack, without distinction, the aged, middle aged and young, and both continue alike, about the same length of time; more severe and fatal in their effects at the onset, than at any subsequent period.

In view of the above facts, taken in connection with numerous others of the same import, we are justified, as it appears to us, in coming to the conclusion that *Cholera and Influenza are so nearly allied as to justify us in assuming that both are produced by the same cause, and that this cause is Ozone.*

Having assumed as we do that *ozone* is the cause of Cholera, we shall now, after refering briefly to a few physiological facts, attempt to explain its mode of action.

According to the views of modern physiologists, the inspired oxygen mingles with the blood in the lungs, without combining chemically with any of its constituents, and remains thus uncombined in a state of diffusion in this fluid, during its passage to, and until it reaches the systemic capillaries, where certain changes are known to take place, such as the disappearance of oxygen, the production of carbonic acid and the generation of animal heat. All the chemico-vital changes constantly going on during the continuance of healthy vital action, take place doubtless in the capillary vessels of each particular part or organ.

It is evident then, that these important changes would not occur, nor could the functions of any organ be properly performed, without a due supply of arterial blood, constituted of its normal constituents, and furnished by the lungs with the proper amount of uncombined oxygen.

Cholera is a disease, the symptoms of which, such as lividity and coldness, shrinking of the skin, stoppage of all the secretions, &c., indicate most clearly the want of healthy action in these vessels. It is absurd to suppose, for instance that in this disease as in health, oxygen and the constituents of the blood and tissues combine chemically, *in the capillaries* since no animal heat is generated *in these vessels* as the result of such combination; yet it is evident from the appearance of the blood in this disease, that such changes do occur, and that arterial is converted into venous blood, more effectually and rapidly even, than in health. The icy coldness pervading the breath, mucous membrane and whole external surface, shows conclusively that no such changes take place either in the pulmonic or systemic capillaries, yet both sides of the heart and all the blood vessels, arteries as well as veins are found to contain, both before and after death, dark venous blood.

These facts, taken in connection with numerous others, which will be referred to hereafter, show as conclusively as the nature of the case will admit, that these changes in the blood, take place *in the arteries, and left side of the heart*, during its passage to, and before it reaches the systemic capilliares. It is evident, that, in health, there exists in these vessels a force, by the action of which, chemical union is effected, between elements, which, though in contact and intimately blended together, do not combine in passing through the heart and large arterial trunks, in which no such force exists.

Now if it can be made to appear that in some diseases, as in cholera, a force equal to, or stronger than this, acts upon the arterial blood during its passage to, and before it escapes the systemic capillaries, causing a chemical combination of its constituents with oxygen, there is no difficulty in explaining how *ozone*, one of the most powerful oxydizing agents known, produces cholera.

When present in the air, it passes into, and is mixed with the arterial blood like pure oxygen and being, as it is, a compound of oxygen and hydrogen; easily decomposes, slight causes effect a separation of its elements and the consequent production in the arteries of new compounds of oxygen and the constituents of the blood, similar to those produced naturally in the capillaries.

During the decomposition of the *ozone*, its oxygen being in the nascent state and consequently more ready to form new combinations, changes the arterial blood to venous, during its passage along the heart and arteries, previous to reaching the systemic capillaries.

This view of the pathology of cholera is sustained both by the symptoms during life, and examinations after death. The burning sensation constantly present at the pit of the stomach, and the insatiable thirst for cold drinks, always manifested by cholera patients, show that so far as their feelings and desires are indicative of their condition, heat must be generated in the heart and large arteries of the chest and abdomen as the result of chemical changes; whilst the entire absence of it, on the surface, at the extremities, and in the mucous membrane lining the cavities, are equally conclusive evidence that no such changes take place in the capillary vessels. Growth nutrition, secretion, and excretion are, to a certain extent, the result of chemico-vital changes in the systemic capillaries, consequent upon the action of uncombined oxygen upon the constituents of the blood and tissues. The almost entire absence, in cholera, of all these vital phenomena, may be explained by assuming, what facts seem to justify, that the blood undergoes a change similar to that from arterial to venous, previous to reaching the capillary vessels of the different organs and tissues.

That the arterial blood in this disease undergoes a change, identical with, or similar to, that which takes place naturally in the systemic capillaries, is evident from numerous facts. Twenty dissections of cholera patients made in Edinburgh by John Lizars Esq., presented indications of this change of the blood, as will be seen by the following quotations. "Brain—This organ was examined in twelve subjects, and in all, the *arteries* and veins of the integuments were distended with dark blood." "Heart—In thirteen the right side was full of dark blood." Aorta—In all it contained more or less dark blood."

Dr. John Ware, in giving a description of the post-mortem appearances in twenty cholera patients, says, "The arteries always contained black blood."

In the Madras report on cholera, will be found the following statements concerning the condition of the blood in the arteries, previous to death. "The temporal artery having been frequently opened, the blood was found to be dark and thick, like venous blood."

The above theory gains additional support from the fact, that the blood secretions and excretions, in cholera, contain an unusual amount of highly oxygenized constituents, in the form of coloring matter, acids, &c. In fact there is nothing, so far as we have been able to judge, in the history, morbid changes, or results of different kinds of treatment in cholera, inconsistent with these views of its cause and pathology.

Facts tending to show that the blood in the arteries of cholera patients, undergoes important changes, effected probably by the influence of oxygen, could be multiplied to almost any extent, but as it seems unnecessary to say more upon this point, we will now pass to the consideration of the most important part of our subject: *The treatment of Cholera.*

Cholera, according to the views and opinions expressed above, is a disease in which the arterial is changed to venous blood, before it reaches the systemic capillaries, or to express our views of its pathology more definitely, its capacity for affording nutriment and supplying animal heat to the different organs and tissues, is impaired or destroyed, as the case may be, as a consequence of the combination of many of its most important constituent with oxygen, under the influence of that most powerful oxydizing agent *ozone.*

If *ozone* is the cause of cholera, as we have endeavored to show, and if the symptoms and morbid changes in this disease are produced by its action as an oxydizing agent upon the constituents of arterial blood, it is evident that the proper remedial agents for this disease are such substances as most effectually aud rapidly destroy *ozone.*

All that class of bodies known as combustibles, possess this power, to a greater or less extent, in proportion to their affinity for oxygen, and are therefore the proper remedies for the disease in question. That they are so, is evident from the fact that highly combustible substances, such as camphor, ether and chloroform, have always been used, and considered by many, as among the most efficient medicines for cholera, This class of medicines has become popular and is used, because experience proves that beneficial results follow their administration; not because we had any just conception of their mode of action: that they are so, no one could doubt, after witnessing the effects recently observed by the writer and numerous others, from the use of sulphur as a remedy for cholera, as first sugested and administered by Dr. J. H.

Bird of this city. The letter upon this subject from him to me as editor of this Journal, has been received too late to appear among the original communications of this number: it is mutually agreed between us, that the most important facts contained in it shall appear, in connection with this, my explanation of its mode of action, and that the next number shall contain a detailed account from him, of cases which have been, or may hereafter be, subjected to the sulphur treatment.

Soon after the reception of the letter referred to above, and after the sulphur treatment had been fully tested by Drs. Bird, Blaney and myself, I was induced, in compliance with the solicitations of numerous citizens who had experienced the beneficial effects following from its use to depart, somewhat, from the course usually pursued by medical men, in reference to newly discovered remedial agents, and make a public announcement of the facts contained in the letter and the results of our experience, in the Chicago Journal of the twenty-ninth of May.

Experience since that time, has convinced us of the propriety of departing somewhat from the course recommended at that time, in using the remedy more frequently and in larger doses. In other respects, our views are as expressed in the letter and embodied in the following extract:

"In one of our recent Medical Journals, an article appeared describing the method of detecting *ozone* in the atmosphere, thus supplying the means of determining whether or not it was present at the very time when cholera was beginning to make its appearance amongst us.

Dr. Bird's experiments as well as those made subsequently by Dr. Blaney and myself from day to day since that time, show that *ozone* is present in our atmosphere, and that the amount is in proportion to the severity of the disease from time to time.

About a week since Dr. Bird determined to try the effects of sulphur upon himself and others, troubled as nearly all have been more or less of late, with uneasy sensations, slight pains &c., in the digestive organs.

The beneficial effects resulting from its use both in his and my practice was such as to convince me at once of its utility in the class of cases described above.

During the last few days Drs. Bird, Blaney and myself have continued to use this apparently simple remedy to the exclusion of nearly all others in all cases of choleraic symptoms. The result has been wonderful. All the premonitory symptoms, such as pain, a sense of fullness, unnatural movements, slight diarrhœa, &c., have uniformly yielded at once to a single dose of three, to four grains of sulphur.

In cases where either cramps, diarrhea or vomiting have been present, and in fact where all these symptoms have existed in conjunction, the use of sulphur in the above named doses every three or four hours, has had the effect to ameliorate at once the patient's condition.

So far as its efficacy has been tested *in the worst stages of collapse*, most satisfactory results have been obtained. In two or three cases of the kind the effect of the remedy has been to bring back pulse to the wrist, restore warmth to the surface, and stop the profuse diarrhœa and vomiting.

In truth, the results obtained so far, have been such as to convince all of us, who have administered it, and witnessed its effects that *if any remedy deserves the appellation, this is a specific for cholera.*

It having been determined to make this public statement, it is expected in return that no hasty conclusions will be made, either for or against what appears to be a proposition to accomplish much by very simple means.

Although the results, so far as obtained, in a short time, and by a few individuals, seem to justify our conclusions, it is hoped that physicians will continue to depend on what they consider the most efficient practice, in bad cases of the cholera, until they shall have tested the matter themselves, and formed their own conclusions; and also, that whatever may be the confidence of individuals in this or any other remedy; they will not depend upon their own judgement in any case, even of slight symptoms, whenever it is possible to consult their physician."

Since the publication of the letter from which the above is an extract, we have become convinced from experience and observation, that sulphur possesses all the powers claimed for it, as a curative agent in cases of pure cholera; but it is liable as we know, to fall into disrepute as a remedy, in the hands of physicians, who, for the want of proper discrimination, look upon and treat, as cholera, during its prevalence, all other diseases of the digestive organs. With regard to the action of sulphur, it may be said, that it is by no means, probable that it is taken into the blood, in the crude state, or that it acts very efficiently in effecting the decomposition of *ozone* in the form of a simple body; on the contrary, the escape of sulphureted hydrogen from the stomach and rectum, and the odor from the surface, always present, whenever sulphur is given to cholera patients, indicate most clearly, that its compounds, such as sulphurous acid and sulphureted hydrogen are found almost immediately after its administration, and therefore that these, of all others the most diffusible and combustible of gases, are the real and efficient agents by which the decomposition and consequent destruction of *ozone* is effected.

If highly combustible gases, such as the sulphureted and carbureted hydrogen, and those constituted in part of ammonia and phosphorus decompose *ozone*, districts in the vicinity of sulphur springs, and of decomposing animal and vegetable matters, ought to be less subject to the prevalence and spread of cholera. That a few sporadic cases may have occurred in the neighborhood of such springs, is not strange; since strangers are constantly visiting such watering places and might be attacked immediately after their arrival, and before they had experienced the beneficial influences of a residence in their vicinity; but so far as can be ascertained by inquiries, assiduously made, of those most likely to be informed upon this point, cholera has never prevailed as an epidemic in sulphur districts. That the gases produced during the decomposition of animal and vegetable matter are preventatives of cholera, is evident from numerous facts, among the most conclusive of which are those obtained by the researches of M. Double in 1832, and embodied in one of the following extracts selected from various sources and offered in conslusion, as additional facts confirmatory of the above views.

M. Double has made a report to the Royal Academy of Medicine on this subject. When the cholera appeared at Blios, a physician of that town saw with fear that the workmen had unpaved the streets for the purpose of cleansing them ; he feared that the removed earth might give rise to fatal or choleric exhalations, an observation which he thought had already been made at Paris. In consequence of which he wrote to the prefect, and the latter transmitted his letters to the minister, who sent them to the Academy to decide the question. Numerous researches were necessary to arrive at this solution : M. Double devoted himself with the greatest care to this subject ; he described all the work of this kind which had been done in Paris since the origin and during the existence of the cholera ; he has followed them from street to street in the different sections of the city ; he has given all their dimensions ; he has pointed out the nature of the several strata of earth which have been dug out, and after the removal of the earth, he has shown that the inhabitants of the streets where the excavations were made, as well as the workmen employed, have been without comparison more healthy than all others. He supports this remark, both by the recent experience of M. Parent-Duchatelet, and by the observation made at Montfaucon, where persons are occupied in the most unhealthy work, and where notwitstanding very few of the workmen are effected. From these facts M. Double conludes that the exhalations which escape from animal matters are not so dangerous as have heretofore been supposed, and that, even during an epidemic, the removal of earth, which increases these exhalations, can be made without danger ; but that this, however, should not restrain the administration, when peculiar circumstances should force it to defer enterprise of this nature. After an animated discussion the Academy decided that the conclusion of this report should not be expressed in terms decidedly affirmative. However, the final drawing up of the report was entrusted to M. Double, and the report and his conclusions were put to the vote and were adopted.—*Archives Generales*, 1832-

"If ozone is the cause of cholera and is the poison which produces the disease, as is alledged by Dr. Bird of Chicago, then it is a simple thing to account for the abscence of cholera in our city at the present time, as well the mild type in which it was exhibited in 1833-4. The sulpher which exists in great quantities in our atmosphere, unites with the ozone and destroys its influence."—*Pittsbnrgh paper, June* 1848.

"If the prevalence of influenza and cholera, be owing to ozone, the vapors of sulpher, or sulphurous gasses, must be protective against it. This is confirmed by, while it explains the immunity of those engaged in, or living near sulpher works."—*Trans. from German, from Heils Zeitschrift.*

"Another circumstance which tends strongly to coroborate the same theory, we have ascertained by inquiring at the city inspectors office. During the cholera of 1832 not one of the many scavengers employed who enhaled an atmosphere tinctured with sulphureted hydrogen, was taken with the disease. Gas manufactories and other establishmens where sulphur is evolved, are also exampt from its attacks."—*New York Tribune.*

During the prevalence of this distemper in '32 I lived in Birmingham, (England) where more Sulphuric Acid is made and used than any other place, perhaps, in the world ; and though all the towns and villages for many miles around were severely afflicted, the disease never reached Birmingham except in one instance of an aged female, and nearly all the physicians there denied that as being cholera.—*Correspondent Ibid.*

I have received yours of this date, [June 13 '49] and have no hesitation in corroborating what you say as to my having visited the Cholera Sheds in 1832-4, for the purpose of seeing that the attendants and nurses did their duty to the poor sufferers or patients. I went among them without fear having impregnated my body with sulphur, owing to my friend Colquhon Sterling, Esq., of Edinbarnet, near Glasgow having assured me that after being 35 years in India, in the Medical Department, and to the head of which he had been raised, he had never known an instance of any person being seized with cholera who had put their bodies into that state; and that even, after the disease had seized them, by dosing them with sulphur and characoal, it generally operated as a cure. *Letter from A. Ferrie to A. Urquhart, Montreal.*

